# Adequate Nutrition in Early Childhood

**DOI:** 10.3390/children10071155

**Published:** 2023-06-30

**Authors:** Agnieszka Kozioł-Kozakowska

**Affiliations:** Department of Pediatrics, Gastroenterology and Nutrition, Jagiellonian University Medical College, Ul. Wielicka 265, 30-663 Kraków, Poland; agnieszka.koziol-kozakowska@uj.edu.pl

For children, receiving adequate nutrition in their first 1000 days of life is vital to ensuring their appropriate growth and preventing the future development of diseases [1]. Metabolic programming through proper diet is one of the ways to prevent future diseases [2].

Breastfeeding and complementary feeding play important roles in determining future health. Breast milk is rich in components that stimulate a baby’s immune system positively from the day it is born, so breast milk is beneficial and should be recommended at least in the first six months of life [3]. The American and European Guidelines recommend exclusive breastfeeding for 6 months and the continuation of breastfeeding until the infant’s first birthday [4,5]. Once milk is no longer sufficient to meet the nutritional needs of a child, a complementary diet should be introduced to prevent deficiencies and ensure the appropriate growth and development of the child. Parts, textures and types of complementary products must be adjusted to the child’s cognitive abilities, health condition, family and local eating habits. Thus, early nutrition establishes important patterns and health trajectories for later life [6]. Both breastfeeding and complementary feeding are among the first dietary exposures a child experiences. Early exposure to different meals may modify and influence a child’s future nutritional preferences [7,8]. Furthermore, children’s preferences for food are related to their patterns of consumption during childhood [9]. The introduction of potentially allergenic foods in the second half of the first year of life is important, thus minimizing the risk of developing food allergies in the future [10]. For this reason, exposure to a variety of food products is very important from early childhood.

The goal of this Special Issue of *Children* is to highlight recent data in the context of child nutrition. This Special Issue publishes a study by Natalie R. JaBaay et al. in a Michigan cohort (USA) of children aged 1–3 whose diet meets basic nutritional recommendations. Breastfeeding rates, fruit and vegetable intake, and the avoidance of added sugars in infancy were all beneficial eating behaviors for the children in the study population, but behaviors related to the restriction of nutrient-poor foods and added sugars in early childhood were not observed. The authors summarize that the area of infant nutrition requires additional public attention and education [11].

Improper diet and a low level of physical activity are the main determinants of the development of the obesity epidemic among children. Nutrition in early childhood, including breastfeeding and complementary feeding, also has a significant impact on the development of obesity [12]. A rapid weight gain trajectory during a child’s first two years of life is associated with an increased risk of the child becoming overweight or obese in adulthood. In 2019, the World Obesity Federation estimated that there would be 206 million children and adolescents aged 5–19 years living with obesity in 2025, and 254 million in 2030 [13]. In a study published in this Special Issue by Zembura et al., it was found that in a sample of Polish preschool children, 16.82% were overweight/obese and 4.49% were obese. Overall, the BMI z-score has been significantly lower since 2017 in the child group; however, in children with overweight and obesity, the BMI z-score was higher in 2017 than in 2007. The z-core of the BMI was positively related to maternal BMI, maternal weight gain during pregnancy, paternal BMI and birth weight [14].

Proper nutrition is needed to halt these negative trends. Establishing healthy dietary patterns in infancy through preschool age may prevent the development of negative health effects in the future and promote a higher quality of life.

I believe this Special Issue contributes to the enhancement of further studies on child nutrition.
